# The County Jails of Georgia

**Published:** 1886-04

**Authors:** 


### Editorial.

### THE COUNTY JAILS OF GEORGIA.

Judge Joel Branham, of the Rome Circuit, in a recent conversation with a *Journal* reporter, during which the subject of county jails was broached, expressed himself in the following vigorous language:

"A great many of the county jails in Georgia are a disgrace to our civilization. They are built with no reference whatever to the comfort of those who may be compelled to occupy them, while, from a sanitary point of view, they are often hardly fit to be occupied by animals, let alone human beings. With few exceptions, they are not furnished with the means of ventilation, and the bad air which the prisoners are compelled to breathe often breeds diseases. Then, again, many of them have no provision for being warmed in cold weather, and much suffering has resulted from this cause during the past winter. The law does not seek to punish a man by freezing him or depriving him of good atmosphere. For this reason, I have made it a point to charge the grand juries wherever I have held court to carefully investigate the jails, and see that they are furnished with proper heating apparatus and the means of ventilation. There should certainly be some state supervision over all prisons, both county and municipal. I have long advocated the appointment of an inspector of such prisons, with plenary powers to enforce all laws pertaining to them."

Col. John R. Towers, the principal keeper of the penitentiary, says that very much of the sickness of convicts in the penitentiary is due to the treatment they receive in the county jails, and that it often takes a considerable time to fully eradicate the seeds of disease there sown from their systems.

According to these statements, there should be a general waking up of the grand juries throughout Georgia, who should see to it that prompt remedies are applied.

The foregoing article, taken from the Atlanta *Journal* of recent date, reproducing the opinion of one of the ablest of our circuit judges, again calls attention to a chronic abuse which for a long period has aroused the disgust and indignation of humane and Christian people.

The sickening cruelties alleged to have been prepetrated in the convict camps have become sufficiently notorious. Newspaper criticisms, legislative investigation, medical supervision and judicial inquiry have, it is hoped, after years of effort, effected some reformation in this department.

The nameless and unwritten tortures of the chain-gang system are now the subject of examination by grand juries, from which it is thought some scheme may be evolved by which the agents of the law may be able to protect society without reviving the practices of the most barbarous tribes of savages.

The bad condition of the county jails to which Judge Branham appropriately calls attention has existed so long, is so widely diffused and the secrets of the prison-house so carefully guarded, that it fails to arrest public attention, and yet these prisons are above all others most entitled to official care and scrutiny; in them are confined alike the accused and the convicted, the innocent and the guilty, and both alike are compelled to breathe for an indefinite time the pestiferous atmosphere of these slaughter-pens, "often hardly fit to be occupied by animals, let alone human beings."

So common is this bad condition of the jails that the lessees of the penitentiary convicts plausibly excuse the fearful mortality in their camps by alleging that the health of the convicted was fatally impaired by their previous confinement. While the death-rate in the camps has been materially lessened by careful effort of the sanitary officers, the testimony of Col. Towers shows "there has been no improvement in the treatment they receive in the county jails."

The county jail of Fulton county is probably one of the best in the State, and for a limited number of prisoners has adequate sanitary arrangements. There are 24 cells 8 by 12 feet, 9 feet between floors, giving to each cell 864 cubic feet of respirable air; 700 cubic feet of respirable air per man, suitably renewed, is as little as will guarantee the health of each inmate, so that 30 prisoners is about the capacity of this jail in the absence of better means for its frequent renewal. A short time ago, from the Federal and State courts there were 144 prisoners crowded into this jail. The result was inevitable and its cause not doubtful. A malignant fever was developed, of which a number of the prisoners died, and but for the prompt action of Judge McKay the mortality would doubtless have been horrible. Sentence upon some was suspended; others were transferred to different prisons, and the disease at once ceased.

No more instructive lesson could be written; no more urgent appeal could be made to all the county authorities, or if these have not now sufficient power, to the General Assembly, to provide a remedy for this shameful evil.

